# Clinical characteristics and outcomes of newly diagnosed patients with HIV‐associated aggressive B‐cell NHL in China

**DOI:** 10.1111/jcmm.17534

**Published:** 2022-09-03

**Authors:** Chaoyu Wang, Jun Liu, Haike Lei, Yu Li, Jian Wu, Bingling Guo, Renzhi Hu, Tingting Liu, Jing Wu, Yao Ding, Chongling Hu, Shunsi Liang, Chunyan Xiao, Xiping Liang, Dehong Huang, Tao Yang, Wenjun Zhang, Zailin Yang, Jieping Li, Yingyu Nan, Qiying Li, Ying Xiang, Zhenhua Li, Yongzhong Wu, Yao Liu

**Affiliations:** ^1^ Department of Hematology Oncology Chongqing University Cancer Hospital, Chongqing Key Laboratory of Translational Research for Cancer Metastasis and Individualized Treatment Chongqing China; ^2^ Chongqing Cancer Research and Control Office Chongqing University Cancer Hospital, Chongqing Key Laboratory of Translational Research for Cancer Metastasis and Individualized Treatment Chongqing China; ^3^ Department of Pathology Chongqing University Cancer Hospital, Chongqing Key Laboratory of Translational Research for Cancer Metastasis and Individualized Treatment Chongqing China; ^4^ Department of Head and Neck Cancer Center Chongqing University Cancer Hospital, Chongqing Key Laboratory of Translational Research for Cancer Metastasis and Individualized Treatment Chongqing China; ^5^ Department of Radiation Oncology Chongqing University Cancer Hospital, Chongqing Key Laboratory of Translational Research for Cancer Metastasis and Individualized Treatment Chongqing China

**Keywords:** BL, Clinical characteristics, DLBCL, HIV, Prognosis

## Abstract

Little is known about the incidence, clinical characteristics and prognostic factors in HIV associated lymphoma as these are less common than HIV‐negative lymphoma in China. Currently, there are no standard guidelines for treatment of these patients. Therefore, we performed a study to analyse the clinical characteristics and outcomes of newly diagnosed HIV‐associated aggressive B‐cell non‐Hodgkin's lymphoma (NHL) patients in Chongqing University Cancer Hospital (CUCH). Totally 86 newly diagnosed HIV‐associated aggressive B‐cell NHL patients in CUCH, southwest China, from July 2008 to August 2021, were analysed. In the entire cohort, median age was 48 years (range, 23–87 years), and more patients were male (87.2%). Most patients had elevated lactate dehydrogenase (LDH) (82.6%), advanced ann arbor stage (80.2%) and high IPI score (IPI score, 3–5) (62.7%) at diagnosis. Median CD4+ T‐cell count at diagnosis was 191/μl (range, 4–1022), 84 patients (97.7%) were on combination antiretroviral therapy (cART) at lymphoma diagnosis. In DLBCL patients, cox multivariate analysis showed that age ≥ 60 (HR = 2.251, 95%CI 1.122–4.516; *p =* 0.012), elevated LDH (HR = 4.452, 95%CI 1.027–19.297; *p* = 0.041) and received less than two cycles of chemotherapy (HR = 0.629, 95%CI 0.589–1.071; *p =* 0.012) were independent risk factors for adverse prognosis based on PFS. Age ≥ 60 (HR = 3.162, 95%CI 1.500–6.665; *p =* 0.002) and received less than two cycles of chemotherapy (HR = 0.524, 95%CI 0.347–0.791; *p =* 0.002) were also independent risk factor for adverse prognosis based on OS. In BL patients, cox multivariate analysis showed that elevated LDH and received less than two cycles of chemotherapy were independent risk factors for adverse prognosis. In the DLBCL group, median PFS times in the received rituximab and no received rituximab groups were not reached and 12 months, respectively (*p =* 0.006). Median OS times were not reached and 36 months, respectively (*p =* 0.021). In the BL group, median PFS times in the received rituximab and no received rituximab groups were not reached and 4.8 months, respectively (*p =* 0.046). Median OS times were not reached and 10.1 months, respectively (*p =* 0.035). Overall, these data indicated that standardized anti‐lymphoma therapy and rituximab administration were significantly associated with improved outcomes in patients with HIV‐associated DLBCL and BL.

## 1 BACKGROUND

According to the 1993 revised classification system for human immunodeficiency virus (HIV) infection, HIV‐associated cancers are divided into acquired immune deficiency syndrome (AIDS)‐defining and non‐AIDS‐defining malignancies based on the coincidence rate among HIV‐infected patients. Non‐Hodgkin's lymphoma (NHL), Kaposi's sarcoma (KS) and invasive cervical cancer are considered AIDS‐defining cancers. Other cancers, including lung cancer, anal cancer, Hodgkin's lymphoma (HL), oral and pharyngeal cancers, hepatocellular carcinoma (HCC), vulvar cancer and penile cancer, are considered non‐AIDS‐defining malignancies in patients with HIV infection.^1^, ^2^ Since 1996, with the wide use of combination antiretroviral therapy (cART), the incidence of malignant cancers in individuals living with HIV has decreased significantly. Since 2017, the incidence of HIV‐associated lymphoma has been higher than that of KS, becoming the highest incidence rate among HIV‐associated cancers in the United States.^2^ Diffuse large B‐cell lymphoma (DLBCL) and Burkitt's lymphoma (BL) are the most common subtypes of HIV‐associated lymphoma.^3^

Treatment of HIV‐associated DLBCL and HIV‐associated BL prior to the advent of cART was frequently complicated by opportunistic infections. Complete remission (CR) rates in the range of 16%–56% and median survival times of 5–8 months were reported regardless of chemotherapy regimen or dose intensity.^4^, ^5^ Since the introduction of cART, studies have suggested better tolerance of chemotherapy and significantly better survival.^6^, ^7^ A phase 2 study assessed CHOP plus rituximab (R‐CHOP) in HIV‐associated B‐cell NHL, revealing a CR rate of 77% and a 2 year‐survival rate of 75%, which suggests that rituximab is beneficial and safe.^8^ In another phase 2 clinical trial of concurrent versus sequential rituximab with the EPOCH regimen in HIV‐associated B‐cell NHL, CR occurred in 21 of 31 evaluable patients (68%, 95% confidence interval [CI] 49%–83%) in the concurrent arm versus 18 of 37 patients (49%, 95%CI 32%–66%) in the sequential arm. The two‐year overall survival rates were 70% (95%CI 57%–83%) and 67% (95%CI 54%–80%) in the concurrent and sequential arms, respectively.^9^ With 5 years of follow‐up, the PFS and OS rates of short‐course EPOCH and dose‐dense rituximab (SC‐EPOCH‐RR) regimen were 84% and 68%, respectively, in HIV‐associated B‐cell NHL.^10^

To date, only a few studies have assessed the clinical features of Chinese HIV‐associated lymphoma patients.^11^, ^12^ Meanwhile, CUCH is the only cancer centre in Western China that treats HIV‐associated lymphoma patients. Herein, we analysed the clinical characteristics and prognosis of 86 newly diagnosed HIV‐associated aggressive B‐cell NHL patients, as the largest cohort reported in China to date.

## 2 METHODS

### 2.1 Patients

**FIGURE 1:**
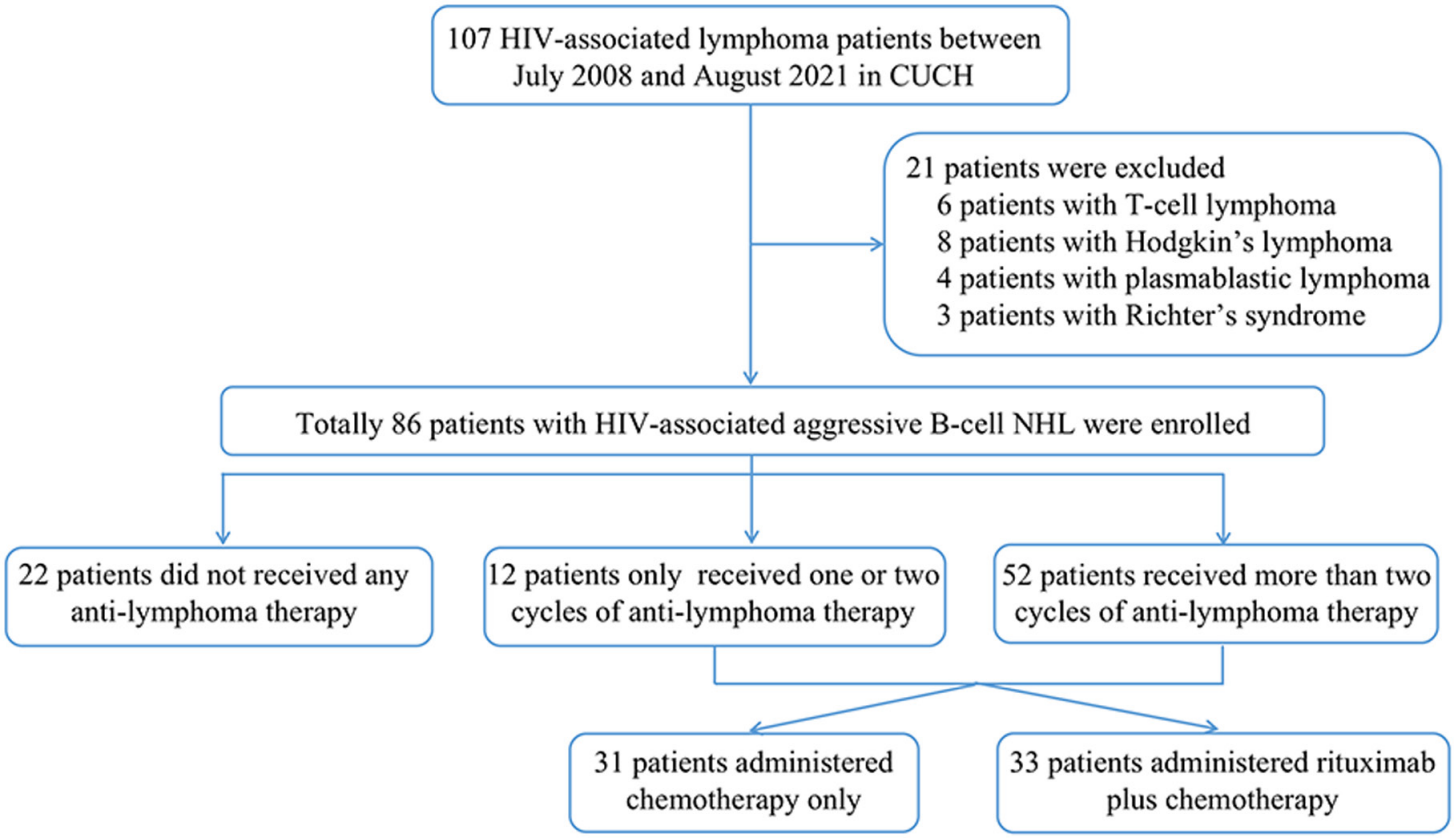
Flow diagram of the study design. Datasets were queried for patients with HIV‐associated lymphoma from July 2008 to August 2021 in Chongqing University Cancer Hospital

All newly diagnosed patients with HIV‐associated lymphoma between July 2008 and August 2021 at CUCH, West China Hospital, were retrospectively reviewed. The exclusion criteria were as follows: T‐cell lymphoma, HL, plasmablastic lymphoma (PBL) and Richter's syndrome. Finally, total of 86 HIV‐associated aggressive B‐cell NHL patients were enrolled, including 63 (73.3%) cases with DLBCL, 20 (23.3%) with BL and 3 (3.5%) with high‐grade B‐cell lymphoma, not otherwise specified (HGBL‐NOS) (Figure 1). The diagnosis was based on 2008 World Health Organization (WHO) classification criteria. All patients were co‐infected with HIV. This study was approved by the institutional review board of CUCH and conducted according to the Declaration of Helsinki (registered at www.chictr.org.cn as #ChiCTR2100054581).

### 2.2 Clinical data analysis

Clinical data, including demographics, HIV transmission route, time from HIV infection to lymphoma diagnosis, CD4 cell count at lymphoma diagnosis, Eastern Cooperative Oncology Group (ECOG) performance status, pathological subtype, the presence of B symptoms, serum lactate dehydrogenase (LDH), serum β_2_‐microglobulin (β_2_‐MG), Ann Arbor stage, International Prognostic Index (IPI) score, extranodal involvement, bone marrow involvement, central nervous system (CNS) involvement, the presence of bulky tumour (maximum diameter ≥7.5 cm) and other related characteristics, including EBV load, EBV‐encoded RNA (EBER), HBsAg and Anti‐HCV IgG, were analysed.

### 2.3 Anti‐lymphoma treatment

Standardized anti‐lymphoma therapy was defined as ≥3 cycles of systemic chemotherapy. Of all 86 HIV‐associated aggressive B‐cell NHL patients, 22 (25.6%) received no anti‐lymphoma therapy because of fear of discrimination and poor financial situation, including 16 DLBCL and 6 BL patients, 12 (14.0%) had one or two cycles of anti‐lymphoma therapy including 6 patients died in the course of treatment, 2 patients received only 2 cycles of chemotherapy before the data were collected and 4 patients gave up further treatment due to poor financial situation and 52 (60.5%) received more than two cycles of anti‐lymphoma therapy**.** Of the 64 aggressive B‐cell NHL cases who received anti‐lymphoma therapy, 31 received systemic chemotherapy only, including 18 DLBCL, 11 BL and 2 HGBL‐NOS patients. This is because Chongqing is located in southwest China, and the economic level of the patients is low. Rituximab was not included in China's health insurance catalogue until 2017, when its widespread use was limited by high prices. And 33 received rituximab plus chemotherapy, including 29 DLBCL, 3 BL and 1 HGBL‐NOS patients. The systemic chemotherapy regimen for the patients was EPOCH. The EPOCH regimen consisted of 96 h continuous infusion of etoposide (50 mg/m^2^/day), doxorubicin (10 mg/m^2^/day) and vincristine (0.4 mg/m^2^/day), plus oral prednisone (60 mg/m^2^/day) from Day 1 to Day 5 and intravenous cyclophosphamide (375 mg/m^2^/day) on Day 5, every 21 days. R‐EPOCH regime consisted of infusion of intravenous rituximab (375 mg/m^2^/day, prior to each chemotherapy) and 96 h continuous infusion of etoposide (50 mg/m^2^/day), doxorubicin (10 mg/m^2^/day) and vincristine (0.4 mg/m^2^/day), plus oral prednisone (60 mg/m^2^/day) from Day 1 to Day 5 and intravenous cyclophosphamide (375 mg/m^2^/day) on Day 5, every 21 days.

### 2.4 Anti‐HIV treatment

In this study, 84 patients (97.7%) were administered cART. Two of them gave up cART for fear of discrimination. cART included two nucleoside reverse transcriptase inhibitors and one nonnucleoside reverse transcriptase inhibitor.

### 2.5 Response assessment

Computed tomography (CT) or ^18^F‐fluorodeoxyglucose positron emission tomography/computed tomography (PET/CT) was performed for radiological evaluation. The 2007 revised Cheson criteria were used to define complete response (CR), partial response (PR), stable disease (SD) and progressive disease (PD).

### 2.6 Statistical analysis

PFS was defined as the time from lymphoma diagnosis to disease progression, relapse or death from any cause. OS was defined as the time from lymphoma diagnosis to last follow‐up or death from any cause. All statistical data were analysed with SPSS version 26 or GraphPad Prism 9. Survival was estimated using Kaplan–Meier curves and compared by the log‐rank test. The Cox proportional hazards regression model was used in multivariate analysis to determine prognostic factors. p < 0.05 was considered statistically significant.

## 3 RESULTS

### 3.1 Patient characteristics

Eighty‐six newly diagnosed HIV‐associated aggressive B‐cell NHL patients were included in this analysis. The baseline clinical features of these patients are summarized in Table 1. The median patient age at diagnosis was 48 years (range, 23–87 years), and 75 patients were male (87.2%). Among the 74 cases with clear transmission routes, 36.5% (27/74) were heterosexual, 54.1% (40/74) were homosexual and 9.5% (7/74) were related to drug injection. So, the main mode of HIV transmission was homosexual intercourse. Of all patients, 52.3% were not aware of their HIV infection until lymphoma diagnosis, 34.9% had an HIV infection history of less than 3 years at the time of lymphoma diagnosis, while only 12.8% had an HIV infection history of more than 3 years. This revealed the shortcomings of HIV screening, and the odds of developing aggressive B‐cell NHL decreased gradually with the prolongation of anti‐HIV therapy.

**TABLE 1:** Clinical characteristics of patients

Baseline characteristics	Total, *n* = 86 (%)	DLBCL, *n* = 63 (%)	BL, *n* = 20 (%)	*p*
Gender				0.784
Male	75 (87.2)	57(90.5)	17 (85.0)	
Female	11 (12.8)	6 (9.5)	3 (15.0)	
Age, y				0.602
Median(range)	48 (23–87)	49 (23–87)	44 (26–69)	
<60	68 (79.1)	48 (71.2)	17 (85.0)	
≥60	18 (20.9)	15 (23.8)	3 (15.0)	
HIV transmission route				0.407
Heterosexual	27 (31.4)	22 (34.9)	5 (25.0)	
Homosexual	40 (46.5)	30 (47.6)	8 (40.0)	
Intravenous drug use	7 (8.1)	4 (6.3)	3 (15.0)	
Others	12 (14.0)	7 (11.1)	4 (20.0)	
Years of HIV infection before lymphoma				0.473
Concurrent	45 (52.3)	36 (57.1)	9 (45.0)	
<3	30 (34.9)	19 (30.2)	9 (45.0)	
≥3	11(12.8)	8 (12.7)	2 (10.0)	
ECOG‐PS				0.199
0–1	72 (83.7)	50 (79.4)	19 (95.0)	
2–4	14 (16.3)	13 (20.6)	1 (5.0)	
B symptoms	29 (33.7)	20 (31.7)	8 (40.0)	0.683
Elevated LDH	71 (82.6)	51 (81.0)	18 (90.0)	0.549
Elevated β_2_‐MG	77 (89.5)	57 (90.5)	19 (95.0)	0.863
Ann Arbor stage				0.798
I/II	17 (19.8)	11 (17.5)	3 (15.0)	
III/IV	69 (80.2)	52 (82.5)	17 (85.0)	
IPI				0.733
0–1	7 (8.1)	5 (7.9)	2 (10.0)	
2	25 (29.1)	18 (28.6)	5 (25.0)	
3	31(36.0)	21 (33.3)	9 (45.0)	
4–5	23 (26.7)	19 (30.2)	4 (20.0)	
Extra‐nodal	27 (31.4)	21 (33.3)	5 (25.0)	0.672
Bulky tumour(≥7.5 cm)	27 (31.4)	17 (27.0)	10 (50.0)	0.101
Bone marrow involvement	20 (23.3)	12 (19.0)	8 (40.0)	0.108
CNS involvement	5 (5.8)	3 (4.8)	2 (10.0)	0.750
CD4 cell count (/μl)				0.785
Median (Range)	191 (4–1022)	203 (4–1022)	177 (25–369)	
<50	8 (9.3)	6 (9.5)	2 (10.0)	
50–199	45 (52.3)	31 (49.2)	11 (55.0)	
200–499	30 (34.9)	23 (36.5)	7 (35.0)	
≥500	3 (3.5)	3 (4.8)	0	
EBV(copies/ml)	*N* = 62	*N* = 44	*N* = 15	0.951
<5 × 10^3^	37 (59.7)	26 (59.1)	9 (60.0)	
≥5 × 10^3^	25 (40.3)	18 (40.9)	6 (40.0)	
EBER positive	30 (34.9)	20 (31.7)	10 (50.0)	0.345
HBV positive	10 (11.6)	8 (12.7)	2 (10.0)	0.747
HCV positive	2 (2.3)	2 (3.2)	0	–
Rituximab‐containing regimen	33/64 (51.6)	29/47 (61.7)	3/14 (21.4)	0.008

The main pathological subtype was the germinal center B‐cell‐like lymphoma (GCB) (74.6%). The median CD4^+^ T‐cell count at diagnosis was 191/μl (range, 4–1022) in the 86 patients, of whom 53 (61.6%) had a CD4^+^ T‐cell count below 200/μl at diagnosis. Most patients (82.6%, 71/86) had elevated LDH, (89.5%, 77/86) elevated β_2_‐MG, and advanced Ann Arbor stage (80.2% 69/86) at diagnosis. High IPI score (3–5) at diagnosis was found in 62.8% (54/86) of patients. Twenty‐seven patients (31.4%) had extranodal involvement. Totally 20 (23.3%) patients had bone marrow involvement. Five (5.8%) patients had CNS involvement, including 3 cases of DLBCL and 2 cases of BL. Totally 27 (31.4%) patients showed bulky tumours, 14 (16.3%) had poor performance status (PS) (ECOG PS 2–4) and 29 (33.7%) had B symptoms at diagnosis. A Ki‐67 proliferation index higher than 80% was observed in 68 cases (79.1%). Of the 86 cases, 16 showed positive CD5 expression by immunohistochemistry (IHC), 56 were negative and 14 were unknown. Among 62 cases with information on EBV status, EBV load was elevated (5 × 10^3^ copies/ml) in 25 (40.3%). And totally 30 patients (34.9%) showed positive EBER by fluorescence in situ hybridization (FISH), including 20 DLBCL patients (31.7%) showed positive EBER, 10 BL patients (50.0%) showed positive EBER. Of all patients, 10 (11.6%) had positive HBsAg and 2 (2.3%) were positive for anti‐HCV antibody. There was no statistical difference in baseline characteristics between patients with DLBCL and BL.

### 3.2 Treatment and efficacy

Among the 86 patients, 22 received no anti‐lymphoma therapy because of fear of discrimination and poor financial situation and 5 had ongoing treatment. Totally 59 HIV‐associated aggressive B‐cell NHL patients were evaluated for best treatment response at the end of treatment. The results indicated an overall response rate (ORR) of 84.7% (50/59), including 24 (40.7%) CR and 26 (30.2%) PR cases. In the 43 evaluable HIV‐associated DLBCL patients, the ORR was 86% (37/43), including 17 (39.5%) CR and 20 (46.5%) PR cases. In the 13 evaluable HIV‐associated BL patients, the ORR was 76.9% (10/13), including 5 (38.5%) CR and 5 (38.5%) PR cases **(**Table 2 **).**

**TABLE 2:** Response evaluation following chemotherapy in HIV‐associated aggressive B‐cell NHL patients

	DLBCL(*N* = 63)	BL(*N* = 20)	HGBL‐NOS (*N* = 3)
Able to evaluate	*N* = 43	*N* = 13	*N* = 3
Overall response rate (ORR)	37 (86.0%)	10 (76.9%)	3 (100%)
Complete response (CR)	17(39.5%)	5 (38.5%)	2 (66.7%)
Partial response (PR)	20(46.5%)	5 (38.5%)	1 (33.3%)
Stable disease (SD)	1(2.3%)	1 (7.7%)	
Progressive disease (PD)	5(11.6%)	2 (15.4%)	
Unable to evaluate	*N* = 20	*N* = 7	
Ongoing treatment	4 (20%)	1 (14.3%)	
No treatment	16 (80%)	6 (85.7%)	

### 3.3 Treatment outcomes

**FIGURE 2:**
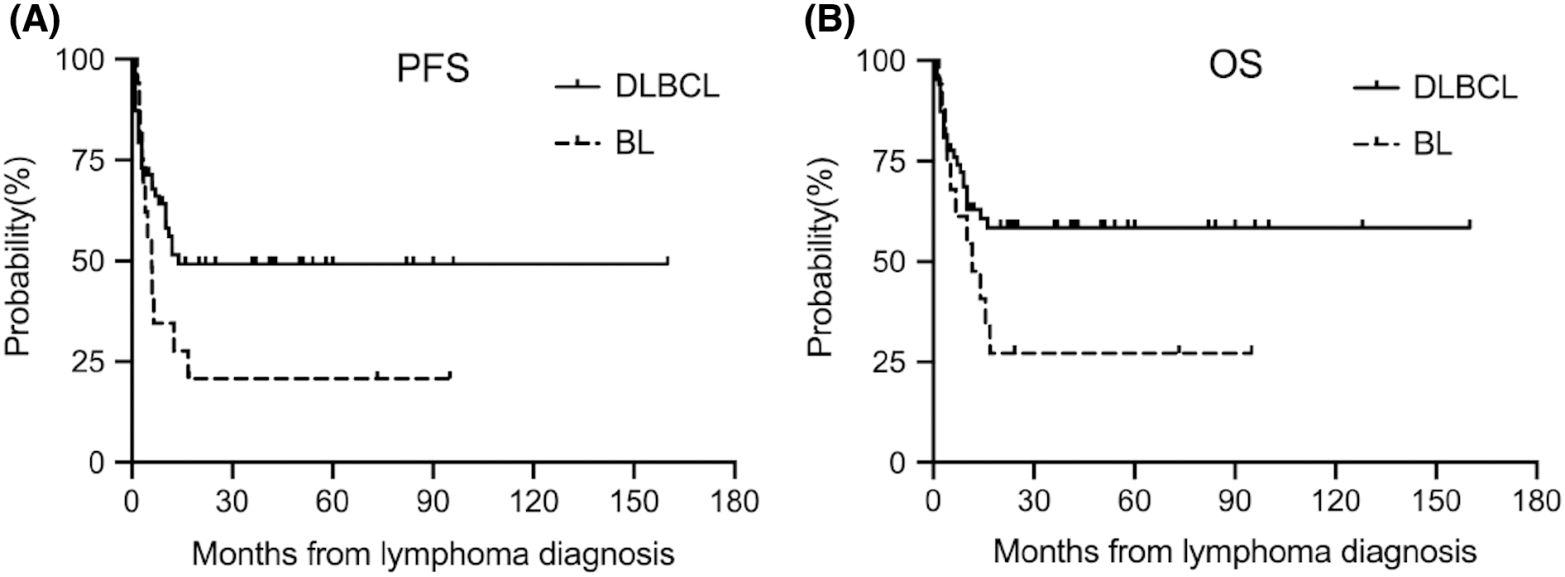
PFS and OS in HIV‐associated aggressive B‐cell patients based on histology subtype. Median PFS times in the DLBCL and BL groups were 18 and 5.9 months, respectively. The overall 2 years PFS rates were 49.2% and 20.7%, respectively (*p* = 0.036) (A). Median OS times in the DLBCL and BL groups were not reached and 11.5 months, respectively. The overall 2 years OS rates were 58.5% and 27.2%, respectively (*p* = 0.047) (B).

Of the 86 HIV‐associated aggressive B‐cell NHL patients. With a median follow‐up of 44.5 months (range, 2–160 months), median PFS and OS were 14 months and 37 months, respectively. The overall 2 years and 5 years PFS rates were 46.8% and 42.1%, respectively. Overall 2 years and 5 years OS rates of 53.6% and 48.8% were observed, respectively. Median PFS times in the DLBCL and BL groups were 18 and 5.9 months, respectively. The overall 2 years PFS rates were 49.2% and 20.7%, respectively (*p =* 0.036) **(**Figure 2A **)**. Median OS times in the DLBCL and BL groups were not reached and 11.5 months, respectively. The overall 2 years OS rates were 58.5% and 27.2% respectively (*p =* 0.047) **(**Figure 2B **)**. These data showed that the survival of HIV‐associated DLBCL was significantly better than that of patients with HIV‐associated BL.

**FIGURE 3:**
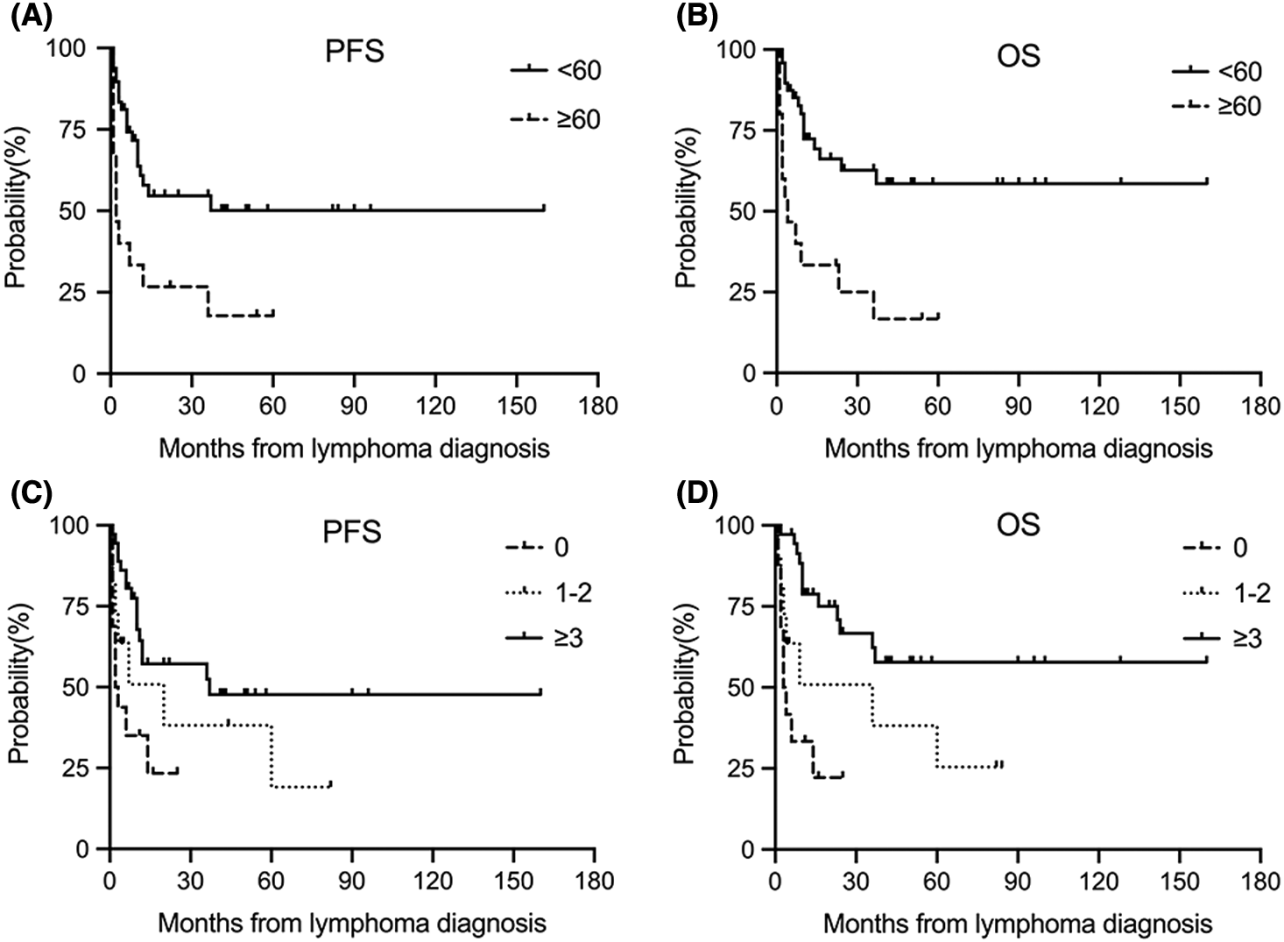
PFS and OS in HIV‐associated DLBCL patients. A and B were PFS and OS for the DLBCL patients based on age. Median PFS times in the age ≥ 60 groups were not reached and 2 months, respectively (*p* = 0.003) (A). Median OS times were not reached and 4 months, respectively (*p* < 0.001) (B). C and D was PFS and OS for HIV‐associated DLBCL patients with or without standardized anti‐lymphoma therapy. Median PFS times in the no received anti‐lymphoma chemotherapy, only received one or two cycles and more than two cycles groups were 2.5 months, 20 months, and 37 months, respectively (*p* = 0.009) (C). Median OS times were 3.5 months, 36 months and not reached, respectively (*p* < 0.001) (D).

In the DLBCL group, median PFS times in the age < 60 and age ≥ 60 groups were not reached and 2 months, respectively (*p =* 0.003) **(**Figure 3A **)**. Median OS times in the age < 60 and age ≥ 60 groups were not reached and 4 months, respectively (*p <* 0.001) **(**Figure 3B **)**. Median PFS times in the no received anti‐lymphoma chemotherapy, only received one or two cycles and more than two cycles groups were 2.5 months, 20 months, and 37 months, respectively (*p =* 0.009) **(**Figure 3C **)**. Median OS times in the no received anti‐lymphoma chemotherapy, only received one or two cycles and more than two cycles groups were 3.5 months, 36 months, and not reached, respectively (*p <* 0.001) **(**Figure 3D **)**. These results demonstrated that age < 60 and standardized anti‐lymphoma therapy were significantly associated with improved outcomes in HIV‐associated DLBCL patients. In the BL group, standardized anti‐lymphoma therapy was significantly associated with improved outcomes.

In this study, there was no patient with primary HIV‐associated central nervous system (CNS) lymphoma. There was no patient along with CNS disease present at diagnosis. All 5 patients routinely administered CNS prophylaxis by intrathecal methotrexate, cytarabine and dexamethasone. A total of five secondary HIV‐associated CNS lymphoma patients, who all had more than two extranodal sites, all died within 24 months after lymphoma diagnosis. These findings suggested very poor survival in secondary HIV‐associated CNS lymphoma.

### 3.4 Prognostic factors

In 63 HIV‐associated DLBCL cases with complete follow‐up data, univariate analysis showed that age ≥ 60 (*p* = 0.001), elevated LDH (*p =* 0.019), advanced stage (*p* = 0.046), high IPI score (3–5) (*p* = 0.032), high ECOG‐PS score (2–4) (*p =* 0.026) and lower CD4 cell count (<200/μl) (*p* = 0.049) and received less than two cycles of chemotherapy (*p =* 0.019) were predictive of worse PFS. Age ≥ 60 (*p <* 0.001), elevated LDH (*p =* 0.012), high IPI score (3–5) (*p* = 0.012), high ECOG‐PS score (2–4) (*p =* 0.002) and received less than two cycles of chemotherapy (*p =* 0.002) were predictive of worse OS. Other factors, including gender, B symptoms, β_2_‐MG, extranodal involvement, bone marrow involvement, bulky tumour and EBV or HBV co‐infection, were not associated with patient prognosis. Cox multivariate analysis showed that age ≥ 60 (HR = 2.251, 95%CI 1.122–4.516; *p =* 0.012), elevated LDH (HR = 4.452, 95%CI 1.027–19.297; *p* = 0.041) and received less than two cycles of chemotherapy (HR = 0.629, 95%CI 0.589–1.071; *p =* 0.012) were independent risk factors for adverse prognosis based on PFS. Age ≥ 60 (HR = 3.162, 95%CI 1.500–6.665; *p =* 0.002) and received less than two cycles of chemotherapy (HR = 0.524, 95%CI 0.347–0.791; *p =* 0.002) were independent risk factor for adverse prognosis based on OS **(**Table 3 **)**. In BL patients, Cox multivariate analysis showed that elevated LDH and received less than two cycles of chemotherapy were independent risk factors for adverse prognosis **(**Table 4 **)**.

**TABLE 3:** Prognostic factor analysis for progression free survival and overall survival in DLBCL patients

Variables	Progression‐free survival				Overall survival			
	Univariate		Multivariate		Univariate		Multivariate	
	*χ*2	*p* value	HR (95% CI)	*p* value	*χ*2	*p* value	HR (95% CI)	*p* value
Gender (F/M)	3.458	0.073			1.803	0.203		
Age (<60/≥60)	10.791	0.001	2.251 (1.122–4.516)	0.012	20.006	<0.001	3.162 (1.500–6.665)	0.002
Stage (I‐II/III‐IV)	4.204	0.046	2.233 (0.720–6.923)	0.234	1.902	0.186		
IPI (0–2/3–5)	6.108	0.032	1.025 (0.450–2.339)	0.943	5.791	0.012	1.253 (0.560–2.805)	0.602
B symptoms	1.212	0.512			0.909	0.444		
ECOG (0–1/2–4)	7.209	0.026	1.621 (0.779–3.372)	0.206	9.983	0.002	1.806 (0.832–3.919)	0.215
CD4 (<200/≥200)	4.246	0.049	0.741 (0.362–1.515)	0.391	2.789	0.084		
β2‐MG	1.105	0.334			2.302	0.149		
LDH	6.672	0.019	4.452 (1.027–19.297)	0.041	5.885	0.012	7.157 (0.951–53.846)	0.065
Extranodal	0.012	0.823			0.076	0.902		
Bulky (>7.5 cm)	0.405	0.542			0.219	0.346		
Chemotherapy cycles (0–2/≥3)	5.958	0.019	0.629 (0.589–1.071)	0.012	11.975	0.002	0.524 (0.347–0.791)	0.002

**TABLE 4:** Prognostic factor analysis for progression free survival and overall survival in BL patients

Variables	Progression‐free survival				Overall survival			
	Univariate		Multivariate		Univariate		Multivariate	
	*χ*2	*p* value	HR (95% CI)	*p* value	*χ*2	*p* value	HR (95% CI)	*p* value
Gender (F/M)	2.401	1.121			1.471	0.225		
Age (<60/≥60)	0.001	0.990			0.400	0.527		
Stage (I‐II/III‐IV)	1.072	0.301			0.085	0.770		
IPI (0–2/3–5)	0.243	0.622			0.250	0.617		
B symptoms	1.717	0.190			1.222	0.269		
ECOG (0–1/2–4)	1.057	0.304			3.315	0.069		
CD4 (<200/≥200)	0.002	0.963			0.361	0.548		
β2‐MG	1.136	0.287			0.946	0.331		
LDH	5.665	0.015	4.769 (1.327–18.307)	0.031	6.966	0.008	5.157 (1.864–20.358)	0.045
Extranodal	0.138	0.710			0.240	0.624		
Bulky (>7.5 cm)	1.129	0.288			2.639	0.104		
Chemotherapy cycles (0–2/≥3)	9.287	0.002	0.531 (0.347–0.974)	0.011	15.704	<0.001	0.334 (0.167–0.671)	<0.001

### 3.5 Rituximab administration and patient outcome

**FIGURE 4:**
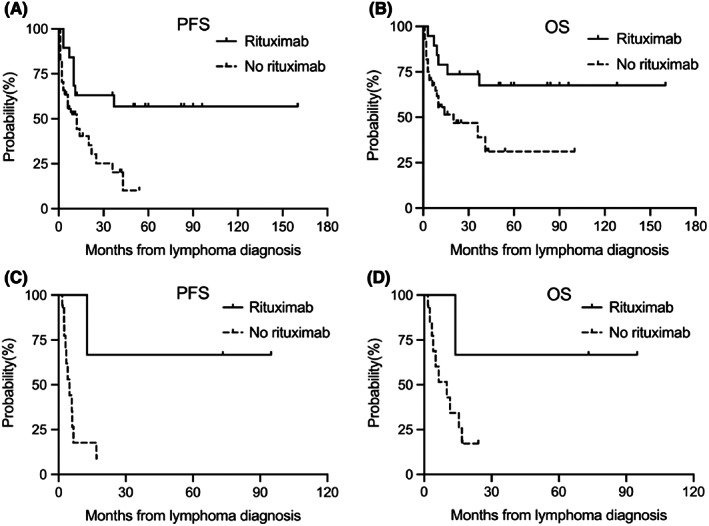
PFS and OS in HIV‐associated DLBCL and BL patients based on receipt of rituximab. In the DLBCL group, median PFS times in the received rituximab and no received rituximab groups were not reached and 12 months, respectively (*p* = 0.006) (A). Median OS times were not reached and 36 months, respectively (*p* = 0.021) (B). In the BL group, median PFS times in the received rituximab and no received rituximab groups were not reached and 4.8 months, respectively (*p* = 0.046) (C). Median OS times were not reached and 10.1 months, respectively (*p* = 0.035) (D)

In the DLBCL group, median PFS times in the received rituximab and no received rituximab groups were not reached and 12 months. The overall 2 years PFS rates were 63.2% and 30.3%, respectively (*p =* 0.006) **(**Figure 4A **)**. Median OS times in the received rituximab and no received rituximab groups were not reached and 36 months. The overall 2 years OS rates were 73.7% and 46.8%, respectively (*p =* 0.021) **(**Figure 4B **)**. In the BL group, median PFS times in the received rituximab and no received rituximab groups were not reached and 4.8 months. The overall 1 year PFS rates were 66.7% and 17.7%, respectively (*p =* 0.046) **(**Figure 4C **)**. Median OS times in the received rituximab and no received rituximab groups were not reached and 10.1 months. The overall 1 year OS rates were 66.7% and 25.8%, respectively (*p =* 0.035) **(**Figure 4D **)**. These data suggested that rituximab administration was significantly associated with improved outcomes in patients with HIV‐associated DLBCL and BL.

## 4 DISCUSSION

In the 40 years since AIDS was first reported, the introduction of cART has greatly improved the prognosis of individuals living with HIV, resulting in significantly increased life expectancy, which is currently close to that of the general population.^13^

After 2017, the incidence of HIV‐associated NHL surpassed KS at 100/100,000 to 300/100,000, making it the most common HIV‐related cancer in the United States.^2^ Due to the particularity of HIV infection, few cancer treatment centres in China treat HIV‐associated lymphoma patients. As the only standard treatment centre for HIV‐associated lymphoma in Western China, we admitted a total of 86 patients with HIV‐associated aggressive B‐cell NHL from July 2008 to August 2021. The present cohort study showed that the median age of HIV‐associated aggressive B‐cell NHL patients was 48 years old (range, 23–87 years). The male‐to‐female ratio was 6.82:1, which indicates a male predilection, in agreement with the overall male‐to‐female HIV incidence rate in China.^14^ Among the 74 cases with clear transmission routes, 36.5% (27/74) were heterosexual, 54.1% (40/74) were homosexual and 9.5% (7/74) were related to drug injection, in agreement with the main transmission routes of HIV infection in China.^14^ It is important to note that 52.3% patients diagnosed with lymphoma were only found to be co‐infected with HIV at the time of diagnosis, indicating that HIV prevalence in this region may be highly underestimated. In this study, 25.6% (22/86) of patients received no anti‐lymphoma‐related therapy, 14.0% (12/86) underwent only 1 or 2 cycles of anti‐lymphoma therapy and 60.5% (52/86) received more than 2 cycles of anti‐lymphoma therapy. This special social problem should also be paid enough attention to by the health and medical departments. Most HIV‐negative NHL patients have painless superficial lymphadenopathy as the primary presentation, and a small proportion of cases have B‐symptoms at onset and early clinical stage.^15^ The initial symptoms of HIV‐associated NHL are more variable, with more than 2/3 of cases being stage III/IV at diagnosis, often accompanied by B‐symptoms and showing more manifestations of extranodal involvement.^16^, ^17^, ^18^, ^19^ The present study revealed up to 80.2% of patients with Ann Arbor stage III/IV, 62.7% with IPI 3–5, and 31.4% with extra‐nodal involvement. These results suggest that HIV‐associated B‐cell NHL are more aggressive and require more urgent treatment. The pathological types of HIV‐associated NHL also differ from those of HIV‐negative individuals. According to a study by Carbone et al., B cells are involved in more than 90%, while T‐cell lymphoma only represents about 3%.^20^ In B‐cell lymphoma, moderate malignant DLBCL and highly malignant BL are the most common types.^11^, ^21^ Our data also showed that DLBCL and BL were the main pathological types. However, this study showed that GCB accounted for 74.6% (47/63) in HIV‐associated DLBCL patients. This is consistent with the conclusion by Reddy et al. that the GCB type is predominant in HIV‐associated DLBCL patients, although the specific mechanism remains unclear.^22^

Currently, there are no standard guidelines for treatment of HIV‐associated lymphoma patients. The common practice is to refer to the guidelines for HIV‐negative lymphoma patients according to different pathological types, and an important treatment strategy is the combined application of cART. In the cART era, combining cART and standardized anti‐lymphoma therapy could significantly improve survival in HIV‐associated NHL patients, with 1 year and 5 years OS up to 66% and 55%, respectively, which are significantly higher than obtained with chemotherapy alone.^23^ The AMC034 trial also suggested that cART combined with anti‐lymphoma therapy may accelerate immune recovery.^24^ This study showed the overall 2 years OS rates in the DLBCL and BL groups were 58.5% and 27.2%, respectively, (*p =* 0.047), which are significantly lower than the OS reported for HIV‐negative lymphoma patients in China.^25^

Because early initiation of cART reduces the risk of developing HIV‐associated NHL, the current treatment recommendation is that antiretroviral therapy should be started as early as possible in patients diagnosed with AIDS.^26^, ^27^ In general, the drugs to be avoided in anti‐lymphoma therapy include those that highly inhibit the bone marrow (e.g. zidovudine) as well as strong cytochrome P450 inhibitors and inducers, which have strong interactions with chemotherapy.^28^ Therefore, we recommend strengthening communication with AIDS specialists and developing personalized cART regimens for the treatment of HIV‐associated NHL.

Rituximab significantly improves the prognosis of HIV‐negative B‐cell NHL patients.^29^ Barta et al. showed that the combination of rituximab and CHOP (R‐CHOP) does not increase mortality due to infectious complications.^30^ Noy and Roschewski also confirmed that rituximab is safe and effective for HIV‐associated BL patients.^31^, ^32^ Coutinho et al. further showed that in the cART era, HIV‐associated DLBCL cases receiving rituximab have better OS (94% vs. 77%, *p* = 0.03) and PFS (78% vs. 64%, *p* = 0.03) compared with HIV‐negative patients.^33^ In our study, in the DLBCL group, median PFS times in the received rituximab and no received rituximab groups were not reached and 12 months (*p =* 0.006). Median OS times were not reached and 36 months (*p =* 0.021). In the BL group, median PFS times in the received rituximab and no received rituximab groups were not reached and 4.8 months (*p =* 0.046). Median OS times were not reached and 10.1 months (*p =* 0.035) . The current data also showed that rituximab combined with systemic chemotherapy significantly prolongs PFS in HIV‐associated DLBCL and BL patients compared with systemic chemotherapy alone as well as OS. However, the phase 3 AMC010 study comparing CHOP and R‐CHOP showed that addition of rituximab to chemotherapy improves the CR rate in HIV‐associated lymphoma patients (58% vs. 47%), although no difference was found in PFS or OS. The reason may be that the benefit gained from disease control is partially offset by increased mortality from infectious complications, especially in patients whose CD4+ cell count below 50/μL, with severe immunodeficiency and those receiving rituximab maintenance therapy. When patients with severe immunodeficiency were excluded, there was no significant difference in infection‐related mortality between the CHOP and R‐CHOP groups.^34^ These studies suggested that patients with HIV‐associated lymphoma may benefit from rituximab. In AMC034, rituximab combined with EPOCH and rituximab administered sequentially after EPOCH were assessed, and the results were compared with CHOP or R‐CHOP in AMC010. The results showed that the CR rate observed with EPOCH alone (followed by rituximab sequentially) (53%) was consistent with those obtained with CHOP and R‐CHOP (57.6% and 47%), while a higher CR rate (69%) was obtained for R‐EPOCH. These results showed that R‐EPOCH is more helpful in prolonging PFS in patients with HIV‐associated DLBCL.^9^ This corroborates Little et al. who assessed 39 patients with HIV‐associated lymphoma treated with EPOCH regimens, with 29 (74%) achieving CR, after a median follow‐up of 53 months, median PFS and OS were 73% and 60%, respectively.^35^ Further AMC075 study found that the CR rate, PFS and OS in HIV‐associated lymphoma are not significantly improved after R‐EPOCH combination with vorinostat, an histone deacetylase (HDAC) inhibitor.^7^

Currently, whether the prognostic risk of HIV‐associated aggressive B‐cell NHL can be assessed using the same model as that of non‐HIV‐infected patients is unclear. Barta SK et al. created a new prognostic index called the AIDS‐related lymphoma International Prognostic Index (ARL‐IPI), which categorizes patients into low‐, intermediate‐ and high‐risk groups.^36^ The ARL‐IPI based on the base‐line characteristics of ECOG performance status, LDH level, stage, number of involved extranodal sites (ENS) and an HIV score that incorporates base‐line CD4 count, HIV viral load and prior history of AIDS. Barta SK et al. found that the ARL‐IPI performed significantly better in predicting OS than the age‐adjusted International Prognostic Index (aa‐IPI).^36^ In this study, cox multivariate analysis showed that age ≥ 60 (HR = 2.251, 95%CI 1.122–4.516; *p =* 0.012), elevated LDH (HR = 4.452, 95%CI 1.027–19.297; *p* = 0.041) and received less than two cycles of chemotherapy (HR = 0.629, 95%CI 0.589–1.071; *p =* 0.012) were independent risk factors for adverse prognosis based on PFS for DLBCL patients. Age ≥ 60 (HR = 3.162, 95%CI 1.500–6.665; *p =* 0.002) and received less than two cycles of chemotherapy (HR = 0.524, 95%CI 0.347–0.791; *p =* 0.002) were independent risk factor for adverse prognosis based on OS for DLBCL patients. In BL patients, cox multivariate analysis showed that elevated LDH and received less than two cycles of chemotherapy were independent risk factors for adverse prognosis.

At the same time, whether HIV infection is a risk factor for increased mortality in HIV‐associated lymphoma remains highly controversial. In B‐cell NHL, HIV infection is no longer an independent predictor of death after controlling for mixed factors such as the frequency of rituximab use.^37^ However, Barnes et al. reported that 14 of the 40 BL patients were HIV positive, and HIV status did not affect the treatment outcome, corroborating Alderuccio et al.^38^, ^39^ Similarly, Noy and colleagues prospectively treated 34 HIV‐associated BL patients with CODOX‐M‐IVAC‐R in AMC048. Although most of the patients had advanced disease and high risk, 1 year PFS and OS were 69% and 72%, respectively. These results were similar to those of HIV‐negative BL patients administered the same regimen.^31^ More prospective clinical studies are needed to establish a prognostic risk assessment system for HIV‐associated lymphoma.

HIV‐associated lymphoma is a highly heterogeneous disease with differences in clinical manifestations, histopathological type and HIV‐infection status. Despite some improvement in the prognosis of HIV‐associated lymphoma with cART combination chemotherapy, there is an urgent need for unified management guidelines to improve survival in this patient population. In addition, autologous haematopoietic stem cell transplant (auto‐HCT), chimeric antigen receptor T (CAR T)‐cell therapy, and new drugs should be assessed more often in relapsed/refractory patients.

In summary, our study suggested that elevated LDH, received less than two cycles of chemotherapy and no received rituximab predicted poor OS and PFS in HIV‐associated DLBCL and BL patients. More high‐quality randomized controlled studies were needed to test our findings.

## AUTHOR CONTRIBUTIONS

**Chaoyu Wang:** Conceptualization (lead); data curation (lead); formal analysis (lead); resources (equal); supervision (equal); validation (equal); writing – original draft (lead); writing – review and editing (equal). **Jun Liu:** Data curation (equal); software (equal). **Haike Lei:** Data curation (equal); supervision (equal). **Yu Li:** Project administration (supporting); validation (supporting). **Jian Wu:** Formal analysis (supporting); validation (supporting). **Bingling Guo:** Methodology (supporting); visualization (supporting). **Renzhi Hu:** Project administration (supporting); software (supporting). **Tingting Liu:** Data curation (equal); software (equal). **Jing Wu:** Project administration (supporting); validation (supporting). **Yao Ding:** Formal analysis (equal); software (equal); validation (equal). **Chongling Hu:** Methodology (equal); writing – original draft (supporting). **Shunsi Liang:** Investigation (supporting); visualization (supporting). **Chunyan Xiao:** Investigation (equal); visualization (equal). **Xiping Liang:** Supervision (equal); writing – review and editing (equal). **Dehong Huang:** Conceptualization (supporting); resources (supporting). **Tao Yang:** Project administration (equal); visualization (equal). **Wenjun Zhang:** Data curation (supporting); software (supporting). **Zalin Yang:** Formal analysis (equal); validation (equal). **Jieping Li:** Investigation (lead); visualization (lead). **Yingyu Nan:** Methodology (equal); validation (equal). **Qiying Li:** Methodology (equal); supervision (equal). **Ying Xiang:** Investigation (equal); visualization (equal). **Zhenhua Li:** Project administration (equal); supervision (equal). **Yongzhong Wu:** Project administration (equal); supervision (equal); validation (equal). **Yao Liu:** Funding acquisition (lead); investigation (lead); supervision (lead); validation (equal).

## FUNDING INFORMATION

The study was supported in part by the National Natural Science Foundation of China (Grant No. 81670100).

## CONFLICT OF INTEREST

The authors declare that they have no competing interests.

## Data Availability

The raw data supporting the conclusions of this article will be made available by the authors, without undue reservation.
